# Foot structure, pain and functional ability in people with gout in primary care: cross-sectional findings from the Clinical Assessment Study of the Foot

**DOI:** 10.1186/s13047-019-0317-2

**Published:** 2019-01-25

**Authors:** Helen R. Petty, Trishna Rathod-Mistry, Hylton B. Menz, Edward Roddy

**Affiliations:** 1 Arthritis Research UK Primary Care Centre, Research Institute for Primary Care and Health Sciences, and Keele Clinical Trials Unit, David Weatherall Building, Keele University, Staffordshire, ST5 5BG UK; 2 School of Allied Health, La Trobe University, Bundoora, Victoria 3086 Australia; 3 Haywood Academic Rheumatology Centre, Staffordshire and Stoke-on-Trent Partnership NHS Trust, Haywood Hospital, Burslem, Staffordshire UK

**Keywords:** Foot, Gout, Primary care

## Abstract

**Background:**

Gout frequently affects the foot yet relatively little is known about the effects of gout on foot structure, pain and functional ability. This study aimed to describe the impact of gout in a UK primary care population.

**Methods:**

A cross-sectional study was nested within an observational cohort study of adults aged ≥50 years with foot pain. Participants with gout were identified through their primary care medical records and each matched on age (±2 years) and gender to four participants without gout. Differences in person-level variables (SF-12 Physical Component Score, Manchester Foot Pain and Disability Index and Short Physical Performance Battery) between gout and non-gout participants were determined using regression models. Differences in foot-level variables (pain regions, skin lesions, deformities, foot posture, and non-weightbearing range of motion) were determined using multi-level regression models. All models were adjusted for body mass index. Means and probabilities with 95% confidence intervals were calculated.

**Results:**

Twenty-six participants with gout were compared to 102 participants without gout (77% male; mean age 66 years, standard deviation 11). Subtalar joint inversion and eversion and 1st metatarsophalangeal joint (MTPJ) dorsiflexion range of motion were significantly lower in the gout participants compared to the non-gout participants. Gout participants were more likely to have mallet toes and less likely to have claw toes compared to non-gout participants. There were no statistically significant differences in person-level variables, foot posture, ankle dorsiflexion range of motion, hallux valgus, pain regions, or skin lesions.

**Conclusions:**

Non-weightbearing range of motion at the subtalar joint and 1st MTPJ was reduced in people with gout. Patients with gout who present with chronic foot problems should therefore undergo appropriate clinical assessment of foot structure.

## Background

Gout is the most common inflammatory arthritis, affecting 2.5% of the UK population [1]. The causal risk factor is hyperuricaemia, leading to the formation of monosodium urate (MSU) crystals in and around the joints, which may result in severely painful acute attacks and a chronic arthropathy. Gout is most common in the small joints, with 43–76% of first episodes occurring in the first metatarsophalangeal joint (MTPJ), but also commonly affects the midfoot and ankle joints [2, 3].

Whilst the predilection of attacks of gout for the foot and ankle is well-recognised, the chronic effects of MSU crystal deposition on foot structure and function have been under-researched. Four small cross-sectional studies performed in New Zealand recruited adults with chronic gout from rheumatology clinics and found higher levels of foot-related pain and disability, reduced peak ankle joint angular velocity and slower walking speed with shorter step and stride lengths, reduced range of motion in the 1st MTPJ dorsiflexion, lower peak plantar pressures in the hallux and had higher pressure time integrals in the midfoot compared to participants without gout [4–7]. Reduced foot and ankle muscle strength in gout has also been demonstrated [8]. However, the generalisability of these findings and whether they can be reproduced in a primary care gout population where disease severity is likely to be milder is unknown.

Therefore, the aim of this study was to describe the effect of gout on the functional and biomechanical characteristics of the foot in a UK primary care population.

## Methods

The Clinical Assessment Study of the Foot (CASF) is a prospective longitudinal cohort study. Adults aged ≥50 years registered at one of four general practices in North Staffordshire, UK were mailed a self-report health survey questionnaire. This questionnaire comprised of 5 main sections: (i) general health; (ii) specific health problems including musculoskeletal co-morbidity and pain; (iii) the presence, duration, location, severity, and impact of foot pain; (iv) demographic and socioeconomic characteristics; and (v) employment. Non-responders were sent reminders after 2 and 4 weeks [9]. Respondents who had indicated they experienced foot pain in the last 12 months and consented to further contact were sent an invitation to attend a research clinic as well as a Participant Information Sheet [9].

At the research clinic, the clinical assessment consisted of a standardised clinical interview and physical examination performed by a clinical assessor. This assessment lasted approximately two hours and involved a physical examination of the feet and simple anthropometric measurements [9].

For this nested analysis, clinic attenders with inflammatory arthritis (rheumatoid arthritis, psoriatic arthritis, or non-specific inflammatory arthritis) identified through their medical records or study radiographs, or those who did not consent to primary care medical record review, or had missing body mass index (BMI) were excluded.

Participants with gout were identified by searching their primary care medical records during the period from 18 months prior to the clinical assessment to 18 months afterwards for specific Read codes for gout. Individuals without a Read code (in the system used across UK general practices, which codes standardised clinical terminology) for gout but who had “gout” mentioned in their consultation free-text were also included if there was mention of a gout attack and/or clinical features (severe pain and inflammation in the joint) consistent with that of gout. The gout participants were then matched on age (±2 years) and gender to four non-gout participants. Participants that could not be matched were excluded.

### Variables

Sixteen variables were chosen from the health survey questionnaire and clinical assessment.

#### Participant characteristics

Participant characteristics used to describe the study sample were socio-economic status based on current or previous occupation; age; gender; any foot pain or ache in the last month, and BMI measured at the clinical assessment.

#### Person-level variables

Variables collected at the person-level were:

i) Physical component score (PCS): calculated using 12 physical component responses from a 36-item health survey, with higher scores indicating better physical function [10].
ii) Foot function and foot pain: calculated from the Manchester Foot Pain and Disability Index (MFPDI) function and pain subscales [9]. It has been shown the function and pain subscales fit the Rasch model thus their transformed scores were used, with higher scores indicating worse function and pain respectively [11].
iii) Short Physical Performance Battery (SPPB): participants were observed performing a series of tests; standing balance test (based on the held side by side stand for 10 s, held semi-tandem stand for 10 s, and held full tandem stand for less than 2 s; 3–9 s and 10 s), time taken to rise from a chair 5 times, and gait speed test (fastest of two timed walks of an 8-ft course at the participants usual walking speed). These were scored individually, and each participant was given an overall SPPB score, with higher scores indicating better lower extremity function [12].

*Foot level variables:* The foot-level variables that were collected in both the left and right feet were:

iv) Hallux valgus: participants were asked to indicate the severity of hallux valgus in each foot using five validated line drawings, increasing in 15° increments. Those selecting one of the three most severe categories in each foot were classed as having hallux valgus [13].
v) Pain regions: participants were asked if they experienced any foot pain or ache lasting more than a day in the last month; if they answered yes, they were then asked to shade a foot manikin, indicating the pain location in their feet. This information was then categorised using a pre-defined marking template [14].
vi) Foot posture index: consisted of a six-item assessment of foot posture, with each item scored between − 2 to + 2, which was then transformed into a single score using Rasch analysis, with lower scores indicating a more supinated foot position and higher scores indicating a more pronated position [15].
vii) Arch index: calculated from ratios of the middle third area to the whole foot area (excluding the toes) ascertained from carbon footprints taken in relaxed bipedal standing [16, 17].
viii) Navicular height: measured in millimetres with a ruler from the floor to the navicular tuberosity with the participant in relaxed bipedal standing, then normalised for foot size by dividing by foot length [17, 18].
ix) Deformity: assessed on physical examination, the palpable presence or absence of deformities on all toes, 1st MTPJ, and lesser toes. MTPJ and interphalangeal joint hyperextension were examined at the 1st MTPJ. For the lesser toes, the type of deformity examined were mallet, hammer, claw and retracted toes [19].
x) Skin lesions: assessed by observation (both plantar and dorsal) of each region of the foot (midfoot, greater toe, and lesser toes) for hyperkeratotic lesions and ulcers.
xi) First MTPJ dorsiflexion non-weightbearing range of motion: measured in degrees using a goniometer, looking at the maximum passive hallux extension with no weight bearing and the ankle relaxed [20].
xii) Subtalar joint non-weightbearing range of motion eversion/inversion: a standardised assessment measuring the participant’s subtalar joint eversion and inversion in degrees using a goniometer [21].
xiii) Ankle dorsiflexion non-weightbearing range of motion with the knee flexed/extended: measured in degrees with an inclinometer during a weight-bearing lunge test [22].

Confounding variables were considered to be age, gender, and BMI [23].

### Statistical analysis

Participant characteristics were described between gout and non-gout participants using appropriate statistics; for continuous variables, the mean and standard deviation and a *p*-value testing for differences between groups via the F-test were reported; for categorical variables, the frequency and percentages and a p-value testing for differences between groups via the Chi-square test were reported.

Person-level variables were compared between gout and non-gout participants using regression models. For continuous variables, linear regression was used and the mean and 95% confidence interval (CI) was reported and significance between the two groups was determined via the F-test. Although the SPPB was treated as a continuous measure, a ceiling effect was present hence tobit regression was used. For binary and ordinal variables, logistic or ordinal regression was used and the probability of having an outcome and 95% CI for gout and non-gout participants were reported and significance between the two groups was determined via the Chi-square test.

Foot-level variables were compared between gout and non-gout participants using random intercept linear or logistic multilevel regression models (as appropriate) to take into account the lack of independence due to clustering of feet belonging to a participant [24]. As described above, results were reported as means or probabilities with 95% CIs and *p*-values.

Although matching on age and gender removed their confounding effects, this resulted in the loss of independence between gout and non-gout participants within a matched group. Therefore, the standard errors were adjusted to take into account this clustering in all analyses. All regression models were also adjusted for BMI.

All analyses were two-tailed and a *p*-value < 0.05 was considered statistically significant. All analysis was performed on STATA v14 (StataCorp, College Station, TX, USA).

## Results

Of the 5109 responders to the health survey questionnaire, 560 participants attended the clinical assessment. Of these 54 were excluded from this analysis, due to lack of consent for medical record review (*n* = 28), inflammatory arthritis (*n* = 24), or missing BMI (n = 2), leaving 506 for sampling. Twenty-six gout participants were identified and then individually matched to four non-gout participants, although two gout participants could only be matched to three non-gout participants each.

Ninety-eight (77%) participants were male and the mean (standard deviation) age was 66.16 (10.77) years. Characteristics of the gout and non-gout participants are presented in Table 1. Age and gender were similar between the groups, indicating successful matching. Mean BMI was similar in both groups. There were more overweight participants in the gout group than the non-gout group, however this was not statistically significant.

**Table 1:** Participant characteristics

Participant characteristics	Non-gout (*n* = 102)	Gout (*n* = 26)	P-value
Age (years): Mean (SD)	66.20 (10.75)	66.04 (11.07)	0.565
Sex: N (%)			
Male	78 (76.47)	20 (76.92)	0.149
Female	24 (23.53)	6 (23.08)	
BMI (kg/m^2^): Mean (SD)	29.11 (4.71)	30.46 (4.43)	0.176
BMI categories: N (%)			
Normal weight < 25 kg/m^2^	20 (19.61)	0 (0.00)	0.207
Overweight 25–30 kg/m^2^	44 (43.14)	16 (61.54)	
Obese ≥30 kg/m^2^	38 (37.25)	10 (38.46)	
Any ache or pain in the feet in the last month: *N* (%)			
Yes	89 (87.25)	20 (76.92)	0.256
Socio-economic status: N (%)			
Higher managerial, administrative and professional	24 (25.53)	4 (16.67)	0.797
Intermediate	15 (15.96)	8 (33.33)	
Routine and manual	55 (58.51)	12 (50.00)	
Ethnicity: N (%)			
White UK/European	97 (97.00)	26 (100.00)	0.371
Afro Caribbean/Asian/African	3 (3.00)	0 (0.00)	
Allopurinol prescription: N (%)			
Yes	–	12 (46.15)	–

*BMI* Body Mass Index, *N* Number of participants, *SD* Standard Deviation

There were no differences in pain, physical function or lower extremity function between the gout and non-gout participants (Table 2). The prevalence of skin lesions, location of foot pain and deformities did not differ between the gout and non-gout participants in either foot, with the exception of hallux valgus, which occurred more frequently in the left foot in the non-gout participants, and mallet toe, which was observed more frequently in the gout participants in the left foot (Table 3).

**Table 2:** Differences in person-level variables between gout and non-gout participants adjusted for BMI

Variables	Non-gout N = 102	Gout N = 26	*P* value
MFPDI pain score: mean (95% CI)	0.02 (−0.27, 0.30)	−0.45 (−1.27, 0.37)	0.272
MFPDI function score: mean (95% CI)	−0.63 (− 0.95, − 0.30)	−0.50 (− 1.41, 0.41)	0.791
SPPB: mean (95% CI)	8.08 (7.29, 8.87)	8.57 (7.08, 10.05)	0.434
PCS: mean (95% CI)	36.50 (34.07, 38.93)	38.87 (33.91, 43.82)	0.343
Side by side stand test: N, probability (95% CI)	100, 0.99 (0.97, 1.01)	25, 0.95 (0.87, 1.04)	0.224
Semi-tandem stand test: N, probability (95% CI)	91, 0.90 (0.84, 0.97)	21, 0.80 (0.64, 0.96)	0.069
Full tandem stand test: N, probability (95% CI)			
Unable to complete or held for < 2 s	22, 0.23 (0.12, 0.34)	6, 0.20 (0.04, 0.36)	0.726
Held for 3–9 s	10, 0.09 (0.04, 0.14)	1, 0.08 (0.03, 0.13)	
Held for 10 s	69, 0.69 (0.57, 0.80)	19, 0.72 (0.54, 0.91)	
Standing balance ability: N, probability (95% CI)			
0 (poor)	1, 0.02 (0.00, 0.04)	1, 0.02 (0.00, 0.04)	0.910
1	9, 0.10 (0.04, 0.16)	4, 0.10 (0.00, 0.21)	
2	12, 0.10 (0.04, 0.16)	1, 0.10 (0.03, 0.17)	
3	10, 0.09 (0.03, 0.14)	1, 0.08 (0.03, 0.14)	
4 (good)	69, 0.69 (0.58, 0.80)	19, 0.70 (0.50, 0.91)	
Time taken to rise from a chair 5 times (seconds): *N*, probability (95% CI)			
Unable to complete	15, 0.14 (0.08, 0.20)	2, 0.11 (0.05, 0.18)	0.454
Slowest risers (15.3, 55.5)	25, 0.24 (0.16, 0.32)	5, 0.21 (0.12, 0.31)	
2nd slowest risers (11.9, 15.2)	19, 0.21 (0.16, 0.26)	7, 0.20 (0.15, 0.25)	
2nd fastest risers (9.3, 11.8)	19, 0.19 (0.12, 0.25)	5, 0.20 (0.13, 0.27)	
Fastest risers (4.1, 9.2)	24, 0.23 (0.13, 0.32)	6, 0.27 (0.13, 0.42)	
Gait speed (seconds): mean (95% CI)	7.24 (5.54, 8.95)	5.81 (2.55, 9.07)	0.436

*BMI* Body Mass Index, *CI* Confidence Interval, *MFPDI* Manchester Foot Pain and Disability Index, *N* Number of participants, *PCS* Physical Component Score, *SPPB* Short Physical Performance Battery

**Table 3:** Foot deformity, pain and skin lesions. Values are N (%)

	Left foot		*P* value	Right foot		*P* value
	Non-gout *N* = 102	Gout *N* = 26		Non-gout *N* = 102	Gout N = 26	
Plantar aspect skin lesion						
Midfoot	51 (50.00)	11 (42.31)	0.553	52 (50.98)	11 (42.31)	0.470
Whole Foot	75 (73.53)	16 (61.54)	0.291	75 (73.53)	17 (65.38)	0.448
Greater toe	57 (55.88)	13 (50.00)	0.577	65 (63.73)	13 (50.00)	0.250
Lesser toes	60 (58.82)	11 (42.31)	0.203	57 (55.88)	11 (42.31)	0.262
Dorsal aspect skin lesion						
Midfoot	3 (2.94)	0 (0.00)	–	6 (5.88)	0 (0.00)	–
Whole Foot	45 (44.12)	7 (26.92)	0.136	47 (46.08)	7 (26.92)	0.097
Greater toe	24 (23.53)	2 (7.69)	0.083	26 (25.49)	3 (11.54)	0.133
Lesser toes	35 (34.31)	6 (23.08)	0.270	35 (34.31)	6 (23.08)	0.290
Pain regions						
1st MTPJ	39 (38.24)	10 (38.46)	0.983	41 (40.20)	10 (38.46)	0.864
Hallux	34 (33.33)	6 (23.08)	0.353	32 (31.37)	11 (42.31)	0.339
Greater toe	50 (49.02)	11 (42.31)	0.541	49 (48.04)	14 (53.85)	0.584
Lesser toes	47 (46.08)	9 (34.62)	0.324	48 (47.06)	9 (34.62)	0.292
Plantar forefoot	32 (31.37)	6 (23.08)	0.433	30 (29.41)	7 (26.92)	0.796
Midfoot	46 (45.10)	12 (46.15)	0.919	47 (46.08)	8 (30.77)	0.216
Medial arch	26 (25.49)	8 (30.77)	0.597	23 (22.55)	5 (19.23)	0.743
Ankle	37 (36.27)	12 (46.15)	0.294	37 (36.27)	8 (30.77)	0.575
Plantar heel	15 (14.71)	5 (19.23)	0.616	17 (16.67)	4 (15.38)	0.887
Hallux valgus	29 (28.43)	1 (3.85)	0.024	31 (30.39)	7 (26.92)	0.745
Deformity						
All toes	61 (59.80)	18 (69.23)	0.420	64 (62.75)	14 (53.85)	0.392
1st MTPJ	10 (9.80)	1 (3.85)	0.377	9 (8.82)	3 (11.54)	0.696
Lesser toes	59 (57.84)	18 (69.23)	0.331	62 (60.78)	14 (53.85)	0.527
Type of deformity for lesser toes						
Hammer	35 (34.31)	10 (38.46)	0.675	31 (30.39)	8 (30.77)	0.966
Mallet	10 (9.80)	10 (38.46)	< 0.001	18 (17.65)	7 (26.92)	0.316
Claw	24 (23.53)	3 (11.54)	0.194	23 (22.55)	3 (11.54)	0.218
Retracted	7 (6.86)	1 (3.85)	0.598	6 (5.88)	1 (3.85)	0.705

*MTPJ* Metatarsophalangeal joint, *N* Number of participants

When combining foot-level variables from the left and right feet (Table 4), the ranges of motion in the subtalar joint inversion and eversion and 1st MTPJ dorsiflexion were lower in the gout participants than the non-gout participants. The probability of having a mallet toe was higher in the gout participants, whereas the probability of having a claw toe was slightly lower in the gout participants compared to the non-gout participants.

**Table 4:** Multilevel regression modelling to account for correlation between feet adjusting for BMI

Variables	Non-gout *N* = 102	Gout *N* = 26	*P* value
Arch index: mean (95% CI)	0.24 (0.23, 0.25)	0.24 (0.22, 0.25)	0.636
Subtalar joint inversion ROM: mean (95% CI)	26.71 (25.09, 28.34)	21.15 (18.23, 24.06)	< 0.001
Subtalar joint eversion ROM: mean (95% CI)	12.12 (11.09, 13.16)	10.00 (8.47, 11.52)	0.010
First MTPJ dorsiflexion ROM: mean (95% CI)	63.09 (59.56, 66.62)	54.42 (47.81, 61.02)	0.035
Ankle dorsiflexion knee extended ROM: mean (95% CI)	61.94 (60.45, 63.42)	62.90 (59.43, 66.37)	0.609
Ankle dorsiflexion knee flexed ROM: mean (95% CI)	51.97 (50.32, 53.63)	55.03 (51.64, 58.42)	0.092
Navicular height: mean (95% CI)	0.18 (0.17, 0.18)	0.17 (0.16, 0.19)	0.922
Foot posture index: mean (95% CI)	2.29 (1.95, 2.64)	2.20 (1.61, 2.79)	0.791
Hallux valgus: probability (95% CI)	0.29 (0.19, 0.39)	0.17 (0.07, 0.27)	0.065
Foot pain regions			
MTPJ: probability (95% CI)	0.39 (0.30, 0.49)	0.38 (0.22, 0.53)	0.831
Hallux: probability (95% CI)	0.32 (0.23, 0.40)	0.32 (0.15, 0.49)	0.987
Greater toes: probability (95% CI)	0.49 (0.39, 0.59)	0.47 (0.31, 0.64)	0.900
Lesser toes: probability (95% CI)	0.47 (0.37, 0.57)	0.33 (0.18, 0.48)	0.149
Plantar forefoot: probability (95% CI)	0.30 (0.20, 0.41)	0.24 (0.12, 0.36)	0.397
Midfoot: probability (95% CI)	0.46 (0.37, 0.54)	0.38 (0.22, 0.54)	0.378
Medial arch: probability (95% CI)	0.24 (0.17, 0.30)	0.25 (0.12, 0.37)	0.901
Ankle pain: probability (95% CI)	0.37 (0.28, 0.45)	0.37 (0.21, 0.54)	0.935
Plantar heel: probability (95% CI)	0.16 (0.13, 0.20)	0.17 (0.14, 0.20)	0.708
Deformity			
1st MTPJ: probability (95% CI)	0.10 (0.04, 0.15)	0.09 (0.00, 0.19)	0.963
All toes: probability (95% CI)	0.62 (0.38, 0.86)	0.64 (0.33, 0.96)	0.898
Lesser toes: probability (95% CI)	0.59 (0.46, 0.72)	0.63 (0.40, 0.86)	0.754
Type of deformity for lesser toes			
Mallet: probability (95% CI)	0.14 (0.09, 0.19)	0.33 (0.15, 0.51)	0.017
Hammer: probability (95% CI)	0.30 (0.17, 0.44)	0.34 (0.09, 0.59)	0.760
Claw: probability (95% CI)	0.18 (0.17, 0.18)	0.17 (0.17, 0.18)	0.041
Retracted: probability (95% CI)	0.06 (0.00, 0.23)	0.05 (0.00, 0.20)	0.927
Plantar aspect skin lesion			
Whole foot: probability (95% CI)	0.74 (0.67, 0.81)	0.65 (0.46, 0.85)	0.350
Greater toe: probability (95% CI)	0.59 (0.51, 0.68)	0.52 (0.34, 0.70)	0.472
Lesser toes: probability (95% CI)	0.57 (0.47, 0.67)	0.43 (0.24, 0.63)	0.275
Midfoot: probability (95% CI)	0.51 (0.41, 0.60)	0.42 (0.21, 0.62)	0.493
Dorsal aspect skin lesion			
Whole foot: probability (95% CI)	0.39 (0.00, 0.89)	0.23 (0.00, 1.10)	0.780
Greater toe: probability (95% CI)	0.24 (0.16, 0.31)	0.11 (0.00, 0.23)	0.100
Lesser toes: probability (95% CI)	0.38 (0.22, 0.54)	0.20 (0.08, 0.31)	0.115

*BMI* Body Mass Index, *CI* Confidence Interval, *MTPJ* Metatarsophalangeal joint, *N* Number of participants, *ROM* range of motion

## Discussion

This study found that people with gout recruited from a UK primary care population have reduced non-weightbearing range of motion in the subtalar joint and the 1st MTPJ compared to those without gout and were more likely to have a mallet toe deformity. In contrast, people with gout were less likely to have a claw toe deformity compared to those without gout. No associations were found between gout and physical function, skin lesions, hammer and retracted toe deformities, or foot posture. There were no consistent differences between gout and non-gout participants in foot pain location, including the 1st MTPJ and hallux, and presence of hallux valgus.

Previous research on functional and biomechanical characteristics of foot disease in participants compared people with severe gout recruited from rheumatology clinics to asymptomatic controls [4, 5]. This differed to our study, where both gout and non-gout participants aged ≥50 years had reported foot pain in the last 12 months and were recruited from primary care. As gout tends to present itself after the third decade of life, it is possible participants have had gout for a number of years. It seems likely that the differences between our findings and those of these previous studies could have arisen from our gout cases having less severe gout (which seems likely in a population recruited from primary care compared with a specialist clinic) and/or our non-gout participants also having foot-related problems. It is therefore noteworthy that despite these differences, people with gout in our study had reduced non-weightbearing range of motion at the 1st MTPJ and subtalar joint. This finding is similar to a previous study which found a larger reduced range of motion by 17.9° (compared to 8.7° in our study) at the 1st MTPJ in gout compared to non-gout participants [7]. We did not, however, replicate certain findings of previous studies [4, 5] that showed that walking speed was significantly slower for gout participants than non-gout participants. This may be due to both gout and non-gout participants having foot pain which is known to have a mediating effect on walking speed [25]. However, the methods used to assess walking speed in our study and other published studies differ, requiring caution when comparing findings of these studies. A previous study [4] and a systematic review [2] also found participants with gout reported higher levels of pain and foot disability than those without gout. In our study, participants with gout reported higher pain levels and foot disability, although this was not statistically significant.

The causes of limited 1st MTPJ and subtalar range of motion are unknown. Both joints are affected by gout and hence it is possible that features of gout such as synovial inflammation or tophus could play a role [7, 26, 27]. A possible alternative explanation for limited range of motion is OA [5], although non-traumatic OA of the subtalar joint is uncommon. First MTPJ OA is common in people with gout [3]. We could not explore these possibilities in the current study and further research is warranted.

The absence of any differences in foot posture measures between the groups may be explained by previous observations suggesting that the flexor tendons and plantar fascia are rarely affected by gout [28]. However, previous studies using dynamic plantar pressure measurements suggest that the foot may function in a more pronated position during walking, as evidenced by increased pressure in the midfoot in people with gout [5]. As we did not measure plantar pressures in our study, we were unable to confirm this in our cohort. There is also the possibility that foot posture index may not have been accurately captured as people could adjust their foot position to offload pain [29]. In people with gout, tophus deposition may significantly alter foot alignment [29].

People with gout were less likely to have hallux valgus in the left foot, which is inconsistent with a previous primary care-based study where hallux valgus was more prevalent in age- and gender-matched control subjects without gout [7, 30]. This could be because the control group in our study included individuals experiencing foot pain, whereas the control group in the previous study [30] were individuals over 30 registered in two general practices. However, there is no pathophysiological reason to expect hallux valgus to preferentially affect the left foot less commonly than the right in people with gout, and this association disappeared when examining hallux valgus across both feet, suggesting a possible spurious finding.

A strength of this study is its primary care setting, ensuring its generalisability and relevance to the majority of people with gout in the UK who are treated entirely in primary care. There are, however, several limitations which merit acknowledgement. Firstly, due to this study being a secondary analysis of data, the number of gout participants was limited, hence reducing statistical power to detect statistically significant differences between the gout and non-gout participants. Considering the large number of variables assessed for differences between gout and non-gout participants, type 2 errors were more likely to occur. However, multi-level regression analysis was performed to account for variables measured for both feet of patients, which increased the power of analysis as opposed to using only one foot as is the case with most other studies. Also, missing data was not an issue with the largest proportion of missing data being 6% for an outcome. Secondly, although the diagnostic gold standard for gout is MSU crystal identification in synovial fluid [31], this is not often performed in primary care, risking misclassification bias. Previous studies, however, have shown that GP diagnosis is reliable [32, 33] with the positive predictive value of a recorded diagnosis of gout in UK medical records being 90% [32]. New classification criteria have been published by ACR/EULAR [34], however publication took place after we completed data collection for this study. Finally, the inability to distinguish which foot was affected by gout limits our ability to investigate the relationship between foot characteristics and the involvement of gout specifically in that foot. All the participants within this study had a history of foot pain, meaning associations between gout and foot characteristics may have been masked. Finally, because this was a nested secondary analysis, clinical characteristics of gout were not available, so we were unable to describe the severity of the gout cases or investigate the effect of severity on the associations observed.

## Conclusions

People with gout demonstrated reduced range of subtalar joint and 1st MTPJ motion compared to those without gout and were more likely to have mallet toe deformities. Further longitudinal large-scale research studies are required to confirm if gout is the cause of these findings and establish how they should be managed. Clinicians should be aware of the impact of gout on non-weightbearing range of motion and undertake appropriate assessment in patients with gout presenting with chronic foot problems.

## Abbreviations

BMI: Body Mass Index
CASF: Clinical Assessment Study of the Foot
CI: Confidence interval
MFPDI: Manchester Foot Pain and Disability Index
MTPJ: Metatarsophalangeal joint
PCS: SF-12 Physical Component Score
SPPB: Short Physical Performance Battery
